# Effects of 1-Methyltryptophan on Immune Responses and the Kynurenine Pathway after Lipopolysaccharide Challenge in Pigs

**DOI:** 10.3390/ijms19103009

**Published:** 2018-10-02

**Authors:** Elisa Wirthgen, Winfried Otten, Margret Tuchscherer, Armin Tuchscherer, Grazyna Domanska, Julia Brenmoehl, Juliane Günther, Daniela Ohde, Werner Weitschies, Anne Seidlitz, Eberhard Scheuch, Ellen Kanitz

**Affiliations:** 1 Institute of Behavioural Physiology, Leibniz Institute for Farm Animal Biology (FBN), D-18196 Dummerstorf, Germany; elisa.wirthgen@googlemail.com (E.W.); mtuchsch@fbn-dummerstorf.de (M.T.); 2 Department of Paediatrics, Institute University Children’s Hospital Rostock, D-18057 Rostock, Germany; 3 Institute of Genetics and Biometry, Leibniz Institute for Farm Animal Biology (FBN), D-18196 Dummerstorf, Germany; atuchsch@fbn-dummerstorf.de; 4 Institute of Immunology and Transfusion Medicine, University of Greifswald, D-17475 Greifswald, Germany; grazyna.domanska@uni-greifswald.de; 5 Institute of Genome Biology, Leibniz Institute for Farm Animal Biology (FBN), D-18196 Dummerstorf, Germany; brenmoehl@fbn-dummerstorf.de (J.B.); j.guenther@fbn-dummerstorf.de (J.G.); ohde@fbn-dummerstorf.de (D.O.); 6 Institute of Pharmacy, University of Greifswald, D-17489 Greifswald, Germany; werner.weitschies@uni-greifswald.de (W.W.); anne.seidlitz@uni-greifswald.de (A.S.); 7 Institute of Pharmacology, University of Greifswald, D-17489 Greifswald, Germany; scheuch@uni-greifswald.de

**Keywords:** indoleamine 2,3-dioxygenase, kynurenine pathway, methyltryptophan, LPS, pig

## Abstract

An enhanced indoleamine 2,3-dioxygenase 1 (IDO1) activity is associated with an increased mortality risk in sepsis patients. Thus, the preventive inhibition of IDO1 activity may be a promising strategy to attenuate the severity of septic shock. 1-methyltryptophan (1-MT) is currently in the interest of research due to its potential inhibitory effects on IDO1 and immunomodulatory properties. The present study aims to investigate the protective and immunomodulatory effects of 1-methyltryptophan against endotoxin-induced shock in a porcine in vivo model. Effects of 1-MT were determined on lipopolysaccharide (LPS)-induced tryptophan (TRP) degradation, immune response and sickness behaviour. 1-MT increased TRP and its metabolite kynurenic acid (KYNA) in plasma and tissues, suppressed the LPS-induced maturation of neutrophils and increased inactivity of the animals. 1-MT did not inhibit the LPS-induced degradation of TRP to kynurenine (KYN)—a marker for IDO1 activity—although the increase in KYNA indicates that degradation to one branch of the KYN pathway is facilitated. In conclusion, our findings provide no evidence for IDO1 inhibition but reveal the side effects of 1-MT that may result from the proven interference of KYNA and 1-MT with aryl hydrocarbon receptor signalling. These effects should be considered for therapeutic applications of 1-MT.

## 1. Introduction

Indoleamine 2,3-dioxygenase 1 (IDO1)-mediated degradation of tryptophan (TRP) along the kynurenine pathway plays a significant role as a counter-regulatory mechanism within the inflammatory immune response [1]. Endotoxin-induced IDO1 activation results in the depletion of TRP and the generation of biologically active TRP metabolites such as kynurenine (KYN), kynurenic acid (KYNA) and quinolinic acid (QUIN), which have important immuno- and neuromodulatory properties [2,3,4,5]. In sepsis, it is proposed that an early hyper-inflammatory phase is followed or overlapped by a prolonged state of immunosuppression, which are potentially life-threatening by the development of septic shock or sepsis-induced immunoparalysis, respectively [6]. Clinical studies have shown an association of enhanced IDO1 activity in the early pro-inflammatory stage of sepsis with an increased disease severity [7] and increased hypotension, which contributes to septic shock due to acute circulatory failure [8]. However, it remains unclear whether the increased IDO1 activity reflects a compensatory response or contributes to overshooting inflammation. During ongoing sepsis, there are indices that a prolonged elevated IDO1 activity increases the risk for poor outcome by provoking the maintenance of a protracted immunosuppressive phase, diminishing immune responsiveness and host defence [7,9,10]. In these clinical studies, the enhanced IDO1 activity was characterized by an accelerated TRP degradation, resulting in an increased KYN/TRP ratio or increased levels of KYN or KYNA. The finding that IDO1 knockout mice are protected against lipopolysaccharide (LPS)-induced septic shock [11] also supports the assumption that an enhanced endotoxin-induced activation of IDO1 provokes detrimental effects within conditions of sepsis. Compared with wild-type mice, IDO1 knockout mice had a higher survival rate within 48 h after an endotoxin shock accompanied by lower levels of the pro-inflammatory cytokines TNF-α and IL-12 and simultaneously higher levels of the anti-inflammatory cytokine IL-10, 4 h after LPS. These results indicate that IDO1 may elicit detrimental pro-inflammatory effects in the early stage of sepsis in contrast to the mainly immunosuppressive effects assumed during ongoing sepsis. In view of these findings, the inhibition of IDO1 activity may be a promising strategy for the prevention of endotoxin-induced shock e.g., after surgery. In patients, who expected a surgery, psychological stress enhanced IDO1 activity already in the pre- and also in the postoperative period, which may contribute to postoperative modulation of immune response [3]. Therefore, the inhibition of IDO1 before a scheduled surgery may reduce the risk for poor outcome associated with an enhanced IDO1 activity during sepsis. Further studies, e.g., on cancer in humans, are investigating the inhibition of IDO1 activity using applications of 1-methyltryptophan (1-MT) as a possible therapeutic approach [12]. 1-MT has been shown to inhibit IDO1 activation in mice, concomitant with a restoration of T-cell proliferation in vitro [13] and of antibacterial defence concurrent with a reduction of depression-like behaviour [3]. Furthermore, the application of 1-MT reduced TNF-α and increased IL-10 in response to endotoxin shock, supporting the findings in IDO1 knockout mice [11].

In a previous study of our group, a pig model for systemic IDO1 activation by intraperitoneal (i.p.) LPS stimulation was established. It resulted in the LPS-induced increase of inflammatory cytokines such as TNF-α and IL-10 followed by the depletion of TRP and an increased generation of TRP metabolites such as KYN and KYNA in plasma, and IDO1 protein expression in blood and various organs [14]. In a subsequent study, the pharmacokinetic properties of 1-MT after subcutaneous (s.c.) application were evaluated and a model for systemic accumulation of 1-MT in plasma and tissues was developed [15]. It is the aim of the present study to examine the protective and immunomodulatory effects of 1-MT against LPS-induced shock in a porcine model, because pigs (*Sus scrofa domestica*) provide obvious advantages with respect to their relevance to human pathophysiology compared with rodents. This species closely resembles humans in anatomy, genetics and physiology and is increasingly used as a model for humans in biomedical research [16,17,18]. We hypothesize that 1-MT may inhibit IDO1 activity, resulting in reduced catalytic degradation of TRP and altered metabolite profile along the KYN pathway after LPS challenge. We suppose that this could result in an attenuation of the LPS-induced innate immune response due to the complex immunomodulatory properties of KYN pathway. Hence, the immune response was characterized by cytokines, haematopoiesis and sickness behaviour. An altered activity of IDO1 by 1-MT and its effects on KYN pathway were validated by the measurement of TRP and the downstream metabolites KYN, KYNA and QUIN.

## 2. Results

### 2.1. 1-MT Modifies TRP and Its Metabolites in Plasma

**Figure 1:**
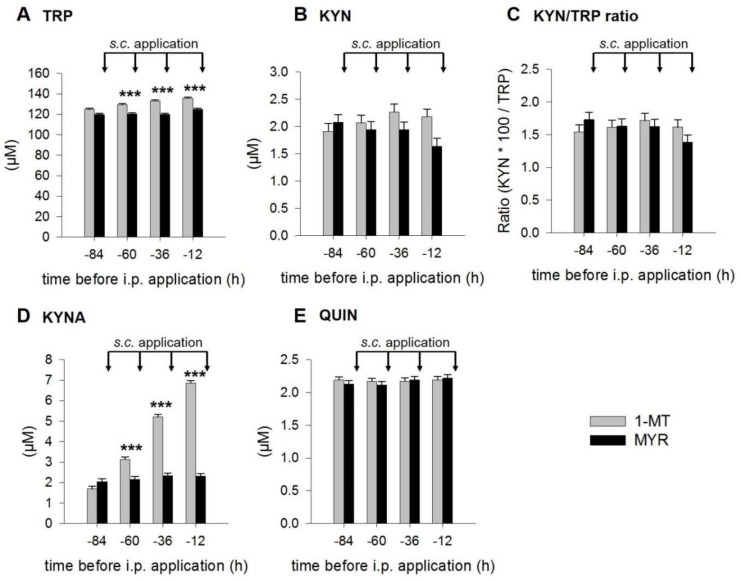
Effects of repeated s.c. 1-MT applications on plasma TRP (**A**), KYN (**B**), KYN/TRP ratio (**C**), KYNA (**D**) and QUIN (**E**) before LPS application. Pre-treatment with MYR was used as a control. TRP and its metabolites were measured using MS/MS and the KYN/TRP ratio was used as a marker for IDO1 activity. The s.c. 1-MT or MYR applications (indicated by black arrows) were injected directly after each blood sampling. The results are presented as LS-means ± SE. Significant differences between the 1-MT- and MYR-pre-treated groups were calculated using the Tukey–Kramer test and are indicated for each time point. 1-MT: *n* = 24; MYR: *n* = 23; *** *p* < 0.001.

According to the experimental design shown in Figure 6, 1-MT was applied 84, 60, 36 and 12 h before the LPS injection (0 h) and 12 h after LPS. Pairwise comparisons revealed no differences in the basal concentrations of TRP, KYN, KYNA, QUIN and the KYN/TRP ratio immediately before the first 1-MT application at the time point −84 h (Figure 1A–E). The repeated s.c. administrations of 1-MT significantly increased plasma concentrations of TRP and KYNA at 60, 36 and 12 h before LPS, whereas KYN, the KYN/TRP ratio and QUIN were not affected by 1-MT.

**Figure 2:**
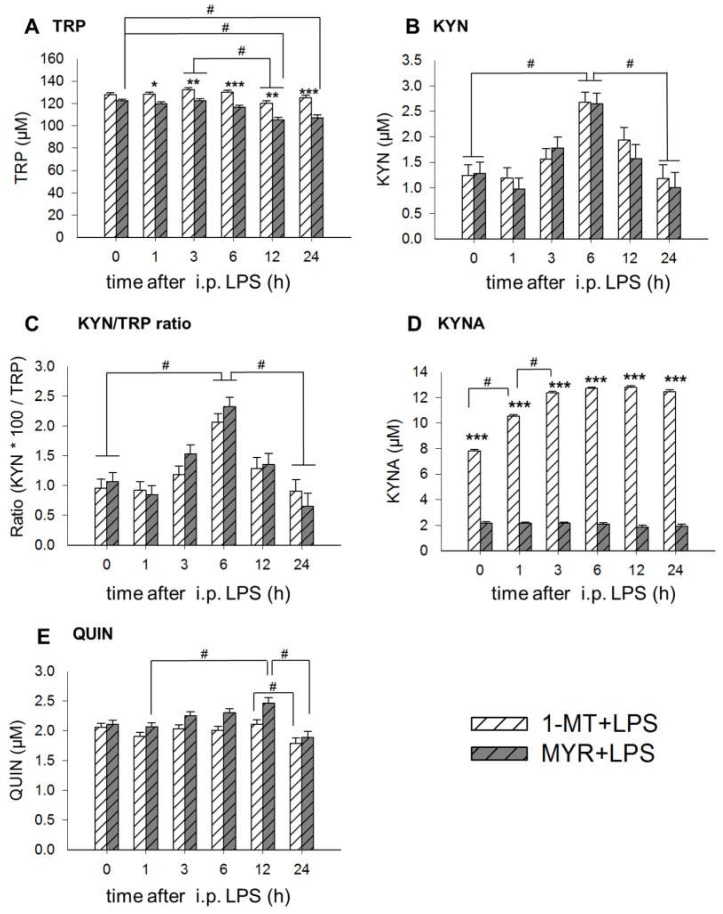
Effects of 1-MT on LPS-induced changes of TRP (**A**), KYN (**B**), KYN/TRP ratio (**C**), KYNA (**D**) and QUIN (**E**) in plasma. As a control for 1-MT-induced effects the excipient MYR was used. TRP and its metabolites were measured using MS/MS and the KYN/TRP ratio was used as a marker for IDO1 activity. LPS was injected i.p. directly after blood sampling at 0 h. The animals received the fifth s.c. 1-MT or MYR injection at 12 h after LPS. The results are presented as LS-means ± SE. Tukey–Kramer procedure was used for all pairwise comparisons. Significant differences are indicated between 1-MT and MYR groups for each time point by asterisks, and between the sampling times by hashes. For the sampling times 0, 1, 3, 6 h: *n* = 11-12/group (total *n* = 47); for time point 24 h: *n* = 5-6/group (total *n* = 23); *** *p* < 0.001, ** *p* < 0.01, * *p* < 0.05, # *p* < 0.01.

Immediately before the LPS application (0 h), KYNA concentrations were significantly increased by 1-MT (Figure 2D, Figure A2D). Likewise, 1-MT generally increased plasma TRP and KYNA after application of LPS or NaCl and reduced plasma concentrations of QUIN (Table 1). Administration of LPS induced a reduction of TRP and an increase of KYN, KYN/TRP ratio, KYNA and QUIN compared to NaCl (Table 1). Significant interactions between 1-MT and LPS effects were found for TRP and KYNA. LPS significantly decreased TRP in Myritol (MYR)-treated controls but not in 1-MT animals and significantly increased KYNA in 1-MT but not in MYR animals (Table 1).

Detailed pairwise comparisons between the different sampling times show that LPS induced a significant decrease of plasma TRP in both 1-MT and MYR pre-treated animals between 3 and 12 h after i.p. application (Figure 2A). However, only in MYR treated animals, TRP was also reduced 12 and 24 h after LPS compared to 0 h. LPS caused a significant increase of KYN and the KYN/TRP ratio at 6 h after administration, which was not affected by 1-MT, respectively (Figure 2B,C). KYNA concentrations were significantly elevated in 1-MT animals at all sampling times (Figure 2D). Further, KYNA concentrations increased in 1-MT animals between 0 to 3 h after LPS, whereas no changes were found in the MYR group. Plasma levels of QUIN increased from 1 to 12 h after LPS in the MYR-treated but not in 1-MT-treated animals (Figure 2E). There was a significant decrease of QUIN from 12 to 24 h after LPS, which was not affected by 1-MT.

**Figure A1:**
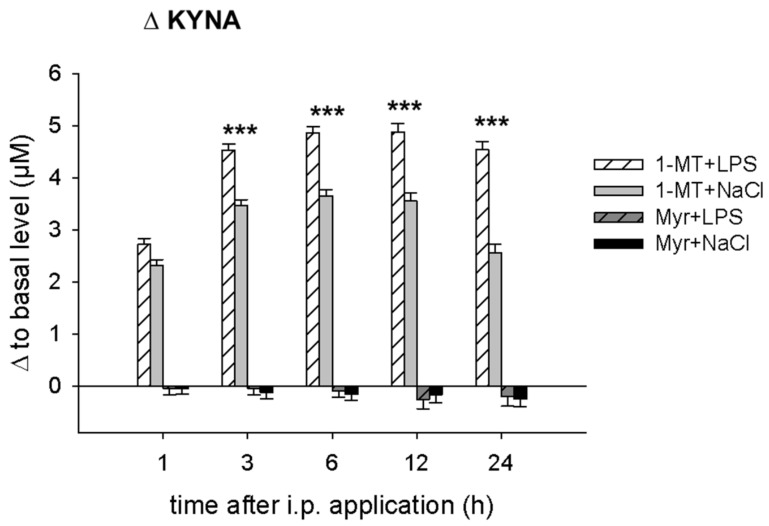
Effect of LPS on the 1-MT-dependent increase of KYNA concentrations in plasma. Treatment with NaCl and pre-treatment with MYR were used as controls. KYNA was measured using MS/MS, and differences in comparison to basal levels (0 h) were calculated to evaluate the LPS effect. The i.p. applications of LPS or NaCl were injected directly after blood sampling at 0 h, respectively. The animals received the fifth s.c. 1-MT or MYR injection at 12 h after LPS/NaCl treatment. The results are presented as LS-means ± SE. Significant differences were calculated using the Tukey–Kramer test and are indicated between LPS and NaCl groups for each time point by asterisks. For time points 0, 1, 3, 6 h: *n* = 11-12/group (total *n* = 47); for time point 24 h: *n* = 5-6/group (total *n* = 23); *** *p* < 0.001.

**Figure A2:**
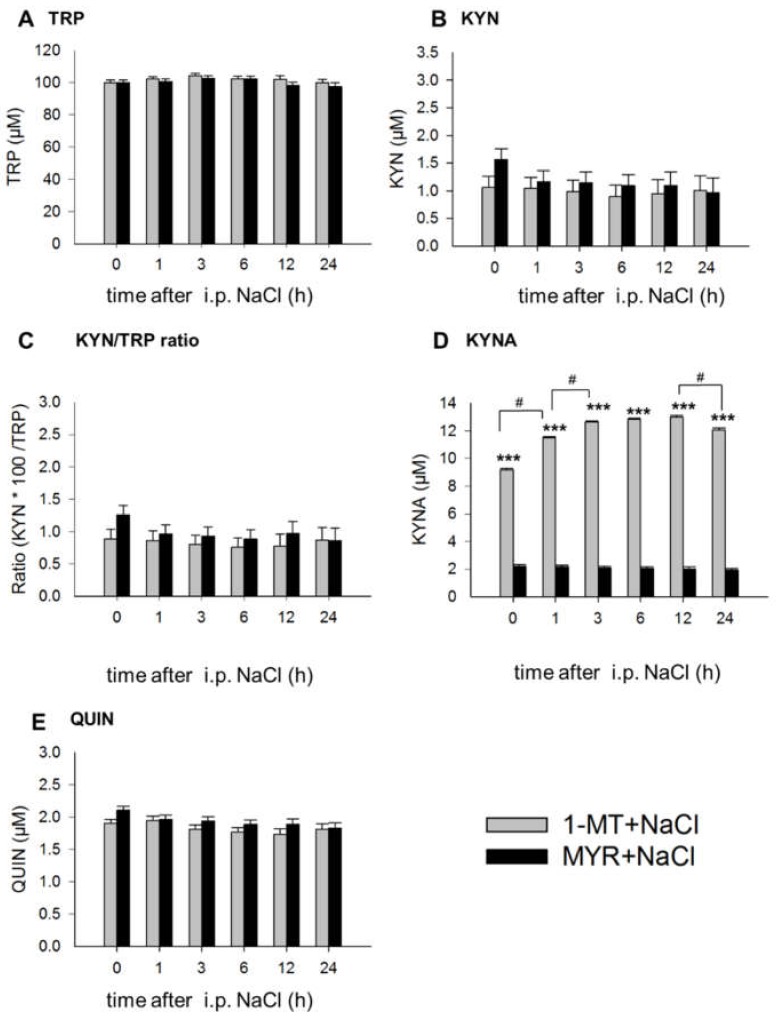
Effect of 1-MT on TRP (**A**), KYN (**B**), KYN/TRP ratio (**C**), KYNA (**D**) and QUIN (**E**) in plasma after i.p. NaCl administration. As a control for 1-MT-induced effects the excipient MYR was used. TRP and its metabolites were measured using MS/MS and the KYN/TRP ratio was used as a marker for IDO1 activity. NaCl was injected i.p. directly after blood sampling at 0 h. The animals received the fifth s.c. 1-MT or MYR injection at 12 h after NaCl. The results are presented as LS-means ± SE. Tukey–Kramer procedure was used for all pairwise comparisons. Significant differences are indicated between 1-MT and MYR groups for each time point by asterisks, and between the sampling times by hashes. For the sampling times 0, 1, 3, 6 h: *n* = 11-12/group (total *n* = 47); for time point 24 h: *n* = 5-6/group (total *n* = 23); *** *p* < 0.001, # *p* < 0.01.

For a better evaluation of the LPS effect on KYNA in 1-MT animals, differences compared with basal levels were calculated and shown as Figure A1. In 1-MT animals, LPS application induced an additional increase in KYNA compared with NaCl-treated 1-MT animals. With the exception of KYNA, the application of NaCl in control animals induced no time-dependent changes of TRP metabolites (Figure A2).

### 2.2. 1-MT Modifies TRP Metabolites in Tissues

TRP, KYN and KYNA were detectable in several tissues and pairwise comparisons of 1-MT vs. MYR and LPS vs. NaCl groups are shown in Table 2. 1-MT significantly increased TRP concentrations in the prefrontal cortex, adrenal gland, muscle, lung, liver, heart, hippocampus, hypothalamus and spleen. KYN was detectable in liver, lung, spleen and hippocampus, but was not affected by 1-MT pre-treatment (Table 2). KYNA was detectable in lung, kidney and liver, and was increased in lung and kidney by 1-MT pre-treatment (Table 2).

Application of LPS induced a significant decrease of TRP concentrations in the amygdala, muscle, lung, liver, heart and kidney and an increase of KYN in lung and spleen (Table 2). A significant interaction between 1-MT and LPS effects was only found for TRP in the adrenal gland. LPS significantly decreased TRP in the adrenal gland of MYR but not of 1-MT animals (*p* < 0.001).

### 2.3. Application of LPS Induces A Reduction of 1-MT in Plasma and Tissue

**Figure 3:**
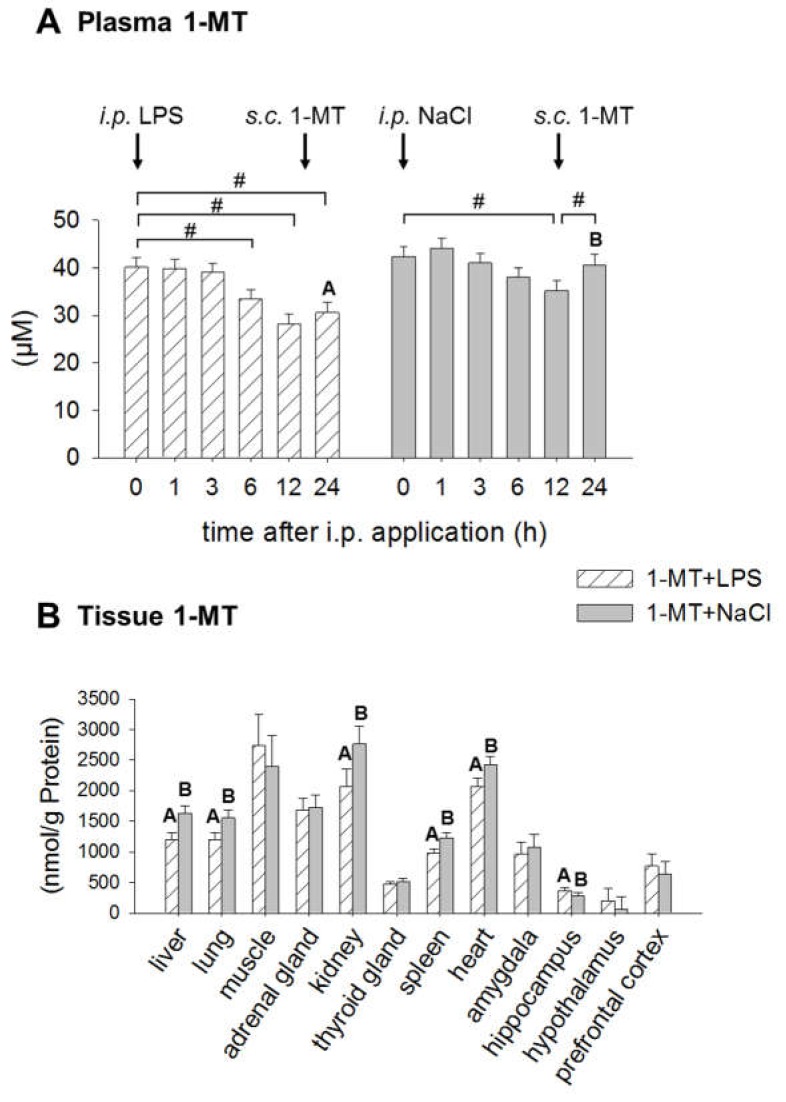
Effects of LPS on 1-MT concentrations in plasma (**A**) and tissues (**B**). Treatment with NaCl was used as a control. MYR-pre-treated animals are not shown due to the absence of 1-MT. 1-MT was quantified using MS/MS. LPS or NaCl (indicated by black arrows) were injected i.p. directly after blood sampling at 0 h. The animals received the fifth s.c. 1-MT injection at 12 h after LPS/NaCl treatment as indicated. The data are presented as LS-means ± SE. In plasma (**A**), significant differences between the time points were calculated using the Tukey–Kramer test and indicated by hashes within each treatment group. Significant differences between LPS and NaCl groups (*p* < 0.05) are indicated using differing letters (A, B). For time points 0, 1, 3, 6 h: *n* = 12/group. For time point 24 h: *n* = 6/group. The data for 1-MT in tissues (**B**) include both sampling times (6 and 24 h, *n* = 12/group) after i.p. application. Significant differences between LPS and NaCl treatment were calculated using the Tukey–Kramer test and are indicated for each tissue, respectively. # *p* < 0.01.

1-MT was not detectable either in plasma or in tissues of MYR animals. In 1-MT animals, plasma 1-MT concentrations decreased 6, 12 and 24 h after LPS compared to concentrations at 0 h (Figure 3A). In the NaCl group, 1-MT was decreased after 12 h, the time of the next 1-MT application, and increased until 24 h. Comparison of both treatments revealed that after 24 h plasma 1-MT was significantly lower in LPS than in NaCl animals. In tissues, the application of LPS also induced a reduction of 1-MT in liver, lung, kidney, spleen and heart (Figure 3B).

### 2.4. 1-MT Modulates Inflammatory Response after LPS Application

**Figure 4:**
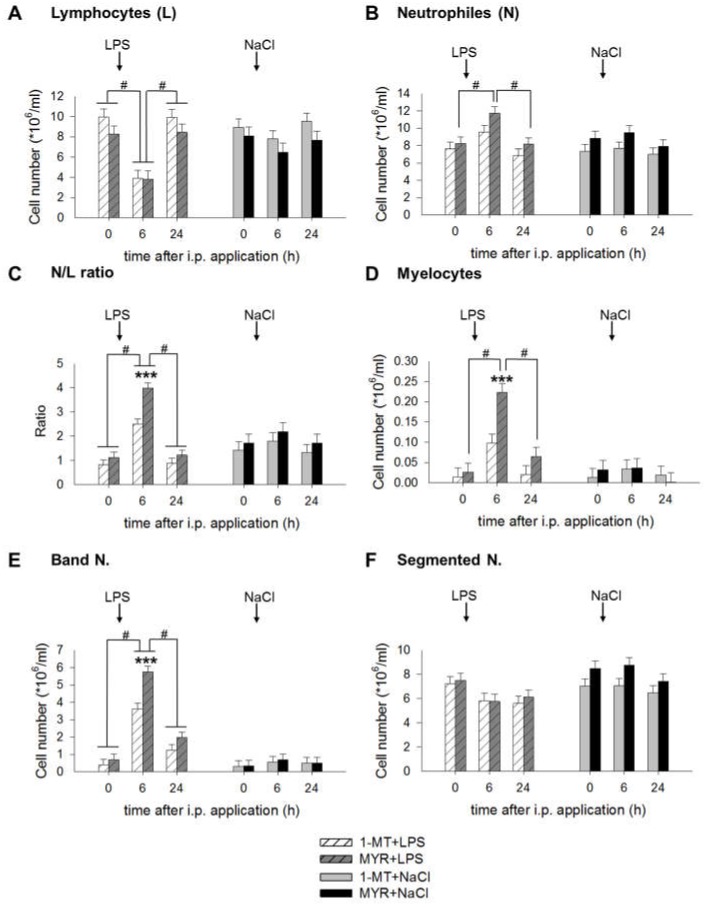
Effects of LPS on the number of lymphocytes (**A**), neutrophils (**B**), N/L ratio (**C**), myelocytes (**D**), band neutrophils (**E**) and segmented neutrophils (**F**) and its interference by 1-MT. Treatment with NaCl and pre-treatment with MYR were used as control groups. LPS or NaCl were injected i.p. directly after blood sampling at 0 h. The animals received the fifth s.c. 1-MT injection at 12 h after LPS/NaCl treatment to prevent a decline in 1-MT levels during the period of the LPS response. The cell number was measured by differential leukocyte counts using a total of 200 leukocytes per animal and time point. All data are presented as LS-means ± SE. Significant differences between 1-MT- and MYR-pre-treated groups were calculated using the Tukey–Kramer test and are indicated for each time point/period by asterisks. Significant differences between the time points are indicated by hashes within each treatment group. For time points 0 and 6 h: *n* = 11-12/group (total *n* = 47); for time point 24 h: *n* = 5-6/group (total *n* = 23); *** *p* < 0.001, # *p* < 0.01.

White blood cells, including lymphocytes and neutrophils were measured before (0 h) and 6 and 24 h after LPS. Summarized over all sampling times, number of lymphocytes was significantly increased in 1-MT animals, irrespective of LPS or NaCl administration (1-MT: 8.3 ± 0.6, MYR: 7.1 ± 0.6 cells × 10^6^/mL, *p* < 0.05). In contrast, the number of neutrophils was generally reduced in 1-MT animals (1-MT: 7.7 ± 0.4, MYR 9.1 ± 0.4 cells × 10^6^/mL, *p* < 0.05). LPS affected the cell number of lymphocytes, neutrophils, myelocytes and band neutrophils and the N/L ratio 6 h after i.p. application. In contrast, NaCl induced no significant changes 6 and 24 h after application. Pairwise comparisons showed no significant differences between 1-MT and MYR pre-treated groups immediately before LPS at time 0 h. LPS induced a decrease of lymphocytes 6 h after i.p. application in both 1-MT and MYR pre-treated animals followed by an increase at 24 h (Figure 4A). An increase of neutrophils was observed in MYR, but not in 1-MT treated animals 6 h after LPS (Figure 4B). The LPS-induced reduction of lymphocytes and increase of neutrophils resulted in an increased neutrophil to lymphocyte (N/L) ratio at 6 h after LPS (Figure 4C) in both 1-MT and MYR pre-treated groups. However, this increase was significantly reduced in 1-MT animals. This was mainly caused by a reduced increase of myelocytes (Figure 4D) and band neutrophils (Figure 4E) in 1-MT animals 6 h after LPS application. Cell number of segmented neutrophils was not affected by 1-MT or LPS (Figure 4F).

**Figure 5:**
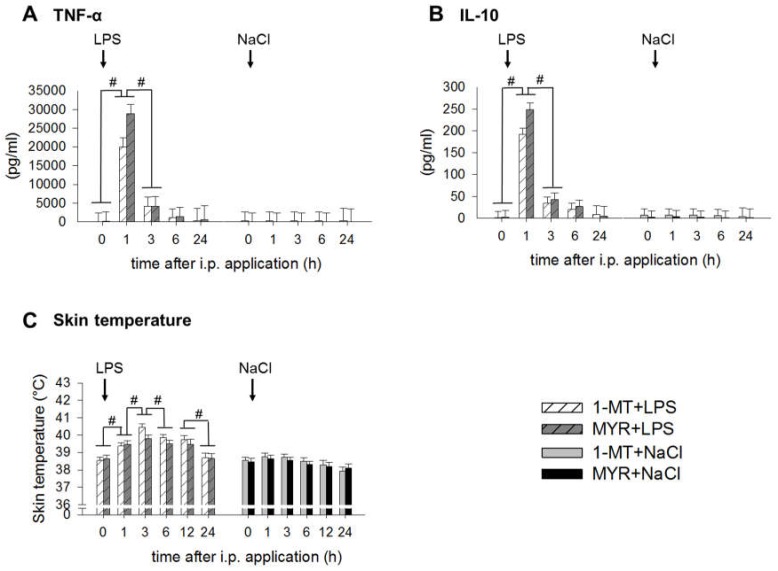
Effects of LPS on plasma TNF-α (**A**), plasma IL-10 (**B**) and skin temperature (**C**) and its interference by 1-MT. Treatment with NaCl and pre-treatment with MYR were used as control groups. LPS or NaCl were injected i.p. directly after blood sampling at 0 h, respectively. The animals received the fifth s.c. 1-MT injection at 12 h after LPS/NaCl treatment to prevent a decline in 1-MT levels during the period of the LPS response. The cytokines TNF-α and IL-10 were quantified by ELISA. Skin temperature was measured using an infrared thermometer directly before blood sampling. All data are presented as LS-means ± SE. For multiple pair-wise comparisons Tukey–Kramer procedure was used. Significant differences between time points are indicated by hashes. For time points 0, 1, 3, 6 h: *n* = 11-12/group (total *n* = 47); for time points 12 and 24 h: *n* = 5-6/group (total *n* = 23); # *p* < 0.01.

The concentrations of TNF-α and IL-10, measured immediately before the LPS application (0 h), were not affected by 1-MT. Treatment with LPS induced a significant increase of plasma TNF-α and IL-10 with the highest concentrations 1 h after LPS and an increase of skin temperature (Figure 5A–C). Pairwise comparisons show that these increases were not affected by 1-MT. Administration of LPS induced sickness behaviour within the 5-h period after administration as shown by inactivity (relative frequency: LPS: 99.97 ± 0.008%, NaCl: 83.36 ± 1.07%, *p* < 0.01) and the occurrence of sickness symptoms (mean number: LPS: 0.69 ± 0.03, NaCl: 0.006 ± 0.01, *p* < 0.001). The severity of sickness after LPS was not affected by 1-MT. However, the pre-treatment with 1-MT increased the inactivity of NaCl-treated control pigs (relative frequency: 1-MT + NaCl: 87.34 ± 1.29%, MYR + NaCl: 78.43 ± 1.67%, *p* < 0.01).

### 2.5. 1-MT Modifies mRNA Expression of Porcine Lung Fibroblasts

**Figure A3:**
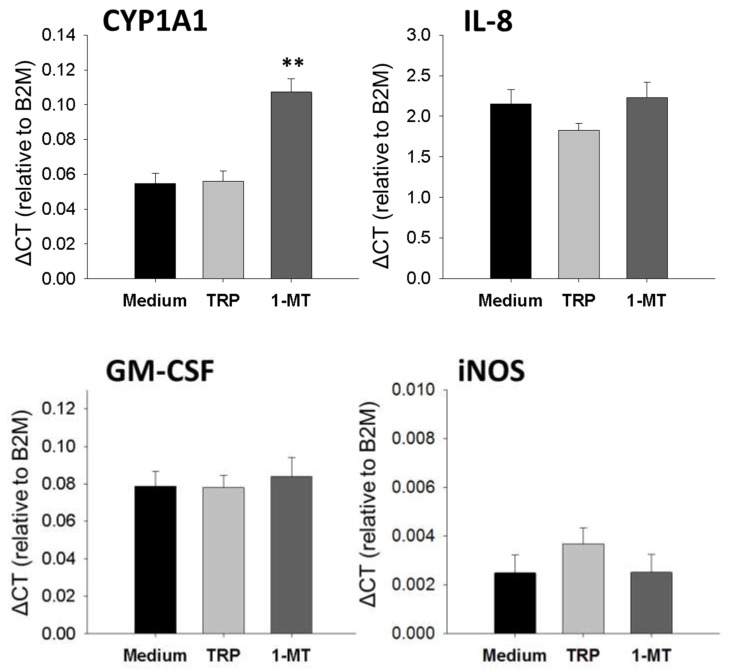
Effects of 1-MT and TRP contained in 1-MT on the mRNA expression of CYP1-A1, IL-8, GM-CSF and iNOS. Porcine lung fibroblasts were incubated with medium (unstimulated) or stimulated with TRP (5 µM) or L-1-MT (100 µM) for 3 h. Data are presented as means ± SE. *n* = 6, ** *p* < 0.01.

A supplementary experiment was conducted to evaluate potential interactions of 1-MT or TRP with aryl hydrocarbon receptor (AhR) or inflammatory signalling pathways in a primary cell culture of porcine lung fibroblasts (See Appendix B.1). The results show that 1-MT significantly increased the mRNA expression of cytochrome P450, family 1, subfamily A, polypeptide 1 (CYP1A1), a marker for AhR activation, compared to the TRP and medium control. The expression of IL-8, granulocyte-macrophage colony-stimulating factor (GM-CSF) and inducible nitric oxide synthase (iNOS) was not affected neither by the 1-MT nor the TRP treatment (Figure A3). TNF-α was expressed on very low levels near or below the detection limit and was not increased, neither by stimulation with 1-MT nor with TRP (data not shown).

## 3. Discussion

In this study, immunomodulatory effects of 1-MT on LPS-induced IDO1 activation were investigated in a porcine model to reveal new insights into the IDO1-mediated effects during the early stage of inflammatory immune response. Our results show that administrations of 1-MT did not diminish the LPS-induced conversion of TRP to KYN, indicating no inhibition of IDO1 activity in vivo. However, 1-MT increased TRP and enhanced the formation of KYNA, a metabolite in the KYN pathway with various immuno- and neuromodulatory functions. In addition, 1-MT caused a modulation of the LPS-induced increase of N/L ratio.

### 3.1. 1-MT-Induced Increases in TRP and KYNA

The results of this study show that 1-MT increased the concentrations of TRP in plasma and several tissues, independently of LPS treatment. This may be the result of reduced TRP degradation or increased availability associated with the administration of 1-MT. In vivo, plasma TRP concentrations are affected by both IDO1 and tryptophan 2,3-dioxygenase 2 (TDO2). Homeostasis of TRP is mainly regulated by TDO2 [19], which is not inhibited by 1-MT [20,21], whereas IDO1 expression is predominantly induced by inflammatory stimuli, such as the cytokines TNF-α or IFN-γ [19]. The increased levels of TRP in our study may reflect a reduced degradation as a result of IDO inhibition due to 1-MT. However, the finding that the LPS-induced increase of KYN was not diminished by 1-MT does not support the assumption of IDO inhibition. According to the manufacturer’s information, 1-MT has a purity of at least 95%. As confirmed by our own HPLC analyses, the L-1-MT contains approximately 5% TRP (data not shown), which corresponds to an s.c. TRP uptake of 0.05 g TRP per day, in addition to a daily dietary uptake of approximately 1.2 to 1.6 g TRP from the digestive tract. Studies in human glioblastoma cells indicate that TRP contamination of commercially available 1-MT (L-isomer) is converted to KYN in vitro [22]. In addition to TRP contamination of 1-MT, a removal of the methyl group of 1-MT by enzymes or chemical processes would result in an increase in TRP concentrations that could not be distinguished from endogenous TRP. Whether TRP contamination of 1-MT and/or demethylation caused the increase in TRP could not be clarified in this study.

Our results show that the production of KYNA, but not KYN, was increased in plasma and tissues in response to 1-MT, independently of LPS. This indicates an enhanced degradation of TRP to one branch of the KYN pathway, which may be a result of increased levels of available TRP. This finding is supported by studies in rats, in which dietary supplementation of TRP induced an increase in urinary excretion of TRP metabolites including KYNA [23]. Furthermore, studies in mice have shown that a repeated application of 1-MT, which also contains low amounts of TRP, results in increased plasma concentrations of KYNA [3]. In recent years, numerous in vivo and in vitro studies have been directed toward the immunomodulatory functions of KYNA. It has been shown that KYNA mediates immunosuppressive effects such as a reduced cytokine response during inflammation in mice [3,5] or acts as a mediator of early recruitment of human monocytes [2]. Moreover, KYNA has been described as a potent natural AhR ligand [24]. AhR signalling is involved in counter-regulatory effects such as reprogramming of the LPS-induced cytokine response and suppression of adaptive immune response [25,26]. Furthermore, AhR is an important regulator of cell physiology and contributes to the proper function of the hepatic, haematopoietic, cardiovascular and immune system [27].

### 3.2. 1-MT Does Not Inhibit the Production of KYN But Is Diminished after LPS Treatment

Previous studies in rodents and in cell cultures showed that 1-MT (L-isomer) reduced the production of metabolites in the KYN pathway and prevented the depletion of TRP [3,13,28,29]. In studies using a recombinant IDO1 enzyme in cell-free assay systems, the L-isomer of 1-MT (K*_i_* = 19 μM/L) was found to be a more potent inhibitor of IDO1 than the D-isomer (K*_i_* > 100 μM/L) [13]. However, the results of this study provide no evidence of an inhibitory effect of the L-isomer of 1-MT on the LPS-induced KYN production in plasma, lung and spleen. Nevertheless, the LPS-induced depletion of TRP was attenuated by the 1-MT-induced general increase in TRP. In vitro studies have shown that the 1-MT-induced increase in TRP may impede the antimicrobial and immunoregulatory functions of LPS-induced TRP depletion [22], facilitating chronic infections due to impaired pathogen growth arrest [12].

One reason for the lack of IDO1 inhibition could be that the concentration of 1-MT was too low to inhibit IDO1 activity in vivo. A phase I trial of tumour patients using 1-MT (D-isomer) as an IDO1 inhibitor has shown that doses higher than 1200 mg 1-MT/patient do not increase peak serum levels [30], indicating a limited accumulation of the applied 1-MT. This finding is in accordance with our previous findings showing that a steady-state 1-MT concentration is already reached after the second 1-MT injection of 1000 mg/animal/day [15], increasing 1-MT to plasma levels similar to those of TRP. Even in lung and spleen, in which 1-MT was approximately 2- to 3-fold higher than the corresponding TRP levels [15], no inhibition of LPS-induced KYN production was detected, assuming that 1-MT did not inhibit IDO1 activity.

It has been shown that 1-MT binds to the ferrous IDO1 enzyme, but the additional methyl group prevents degradation along the KYN pathway due to steric effects [20]. The results of the present study reveal that LPS induces a reduction of 1-MT in plasma and several tissues, which might be a result of an increased urinary clearance and/or of catalytic degradation. There are few indices showing that both IDO1 and TDO2 are able to catalyse 1-MT (L-isomer) with low affinity (*K*_m_ = 150 µM) in comparison to TRP (*K*_m_ = 7 µM), resulting in the generation of KYN or methyl-KYN [31,32]. However, the results of our study show no evidence of additional production of KYN after LPS treatment. Furthermore, in the subsequent HPLC analysis, using α-methyl-KYN as a reference standard, no methyl-KYN was detectable in samples after LPS treatment (data not shown).

### 3.3. 1-MT Mediates Immunomodulatory Effects

In the experimental design of this study, IDO1 activation was induced by LPS resulting in an inflammatory response that provoked an increase in pro- and anti-inflammatory cytokines such as TNF-α or IL-10. Contrary to findings in mice [11], no significant effect of 1-MT on the plasma cytokine response or sickness severity was found. However, the results show that 1-MT induced a modulation of LPS-induced immune response. 1-MT reduced the increase in the immature myelocytes and band neutrophils in response to LPS, indicating a suppression of adequate neutrophil maturation. This may also explain the generally reduced number of neutrophils by 1-MT. Furthermore, 1-MT reduced the LPS-induced increase of the N/L ratio, which is used as an early marker of acute inflammation [33,34]. These results support findings in human cancer and dendritic cells, revealing that 1-MT has transcriptional effects that may promote immunosuppressive effects [35,36].

1-MT enhanced the inactivity of the NaCl-treated animals and elevated number of lymphocytes, which might reflect a response to a repeated inflammatory stimulation [37,38]. In vitro, there are indications that 1-MT interferes with TLR signalling in dendritic cells, independently of IDO1 activity [35]. Furthermore, there are indications in human and mouse cells that 1-MT itself acts as an agonist for AhR, enabling the transcriptional expression of AhR-specific target genes [39], which may affect inflammatory pathways. Results of the supplementary experiment show that 1-MT significantly increased the mRNA expression of CYP1A1, which is described as a sensitive marker for AhR activation [40], thus confirming the findings in mouse and human cell lines [39]. In addition, no increased expression of inflammatory mediators such as IL-8, GM-CSF, TNF-α and iNOS was detected on transcription levels after 1-MT incubation. Further, elevated TRP levels, simulating the 5% of TRP contained in 1-MT, had no significant effects on gene expression. Our findings that 1-MT itself acts as an AhR ligand and increases plasma and tissue concentrations of the endogenous AhR ligand KYNA in vivo should be taken into account when using 1-MT for therapeutic applications and require further investigation.

## 4. Materials and Methods

### 4.1. Animals

Male German Landrace pigs (*n* = 96), bred and raised in the experimental pig unit of the Leibniz Institute for Farm Animal Biology, were used in two experiments. All pigs received standard processing (oral iron supplementation and castration) within the first three days of life. At the beginning of the experiments, the pigs were seven weeks old and weighed between 12 and 18 kg. The pigs were fed a commercial diet and had free access to water. All procedures involving animal handling and treatment were in accordance with the German animal protection law and were approved by the responsible authority (Landesamt für Landwirtschaft, Lebensmittelsicherheit und Fischerei, Mecklenburg-Vorpommern; Rostock; Germany; LALLF M-V/TSD/7221.3-1.1-027/10; 02 Juni 2010).

### 4.2. Experimental Design

**Figure 6:**
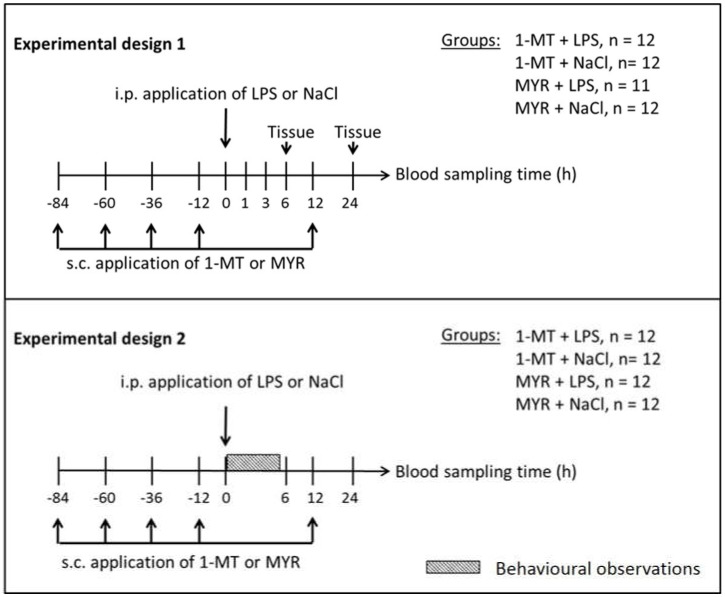
The effects of 1-MT application on TRP metabolism and LPS-induced immune response were investigated in two experiments. In both experiments, 1-MT and its control MYR were repeatedly injected subcutaneously (s.c.) as indicated, aiming at maximum levels of 1-MT at the time of LPS or NaCl injections at 0 h according to the 1-MT kinetics. In experiment 1 (*n* = 47), blood samples were taken before and at 0, 1, 3, 6, 12 and 24 h after LPS/NaCl application and tissue was sampled after 6 h (*n* = 24) and after 24 h (*n* = 23). In experiment 2 (*n* = 48), blood samples were taken before and at 0, 6, 12 and 24 h after LPS/NaCl application and behaviour was observed in the period from 0 to 5 h. Both experiments were conducted in three replicates with 15 or 16 animals each.

The effects of 1-MT application on TRP metabolism and endotoxin-induced immune response were investigated in two experiments (Figure 6). The first experiment focused on 1-MT-induced effects on blood and tissue parameters before and during the 24 h period after a single endotoxin application (*n* = 48). In the second experiment, the influence of 1-MT on LPS-induced sickness behaviour and haematopoiesis was investigated (*n* = 48). Both experiments were conducted in three replicates with 16 animals each. One week prior to the commencement of the experiments, animals were housed in single pens. In each experiment, half of the animals received daily administrations of 1 g 1-MT (L-isomer, purity 95%; Sigma-Aldrich, Deisenhofen, Germany) over a period of five days. The dose was selected according to a preceding experiment. At the time of LPS injection (0 h) steady state plasma levels of 1-MT were achieved and 1-MT was accumulated in tissues at a level equal to or higher than TRP (for details and pharmacokinetics see Wirthgen et al., 2016 [15]). Injections were given s.c. in the popliteal fossa of the hind legs and split in two doses of 0.5 g of 1-MT in 4.0 mL of triglyceride solution Myritol^®^318 (MYR) (Caesar und Loretz GmbH, Hilden, Germany), which was used as an excipient. Control animals received an equivalent volume of the MYR solution. Injections of MYR or 1-MT were given at 08:00 p.m. on five consecutive days (84, 60, 36 and 12 h before, and 12 h after the i.p. LPS/NaCl application). Health status was continuously checked by visual inspection twice daily, and the daily feed uptake was measured. Repetitive s.c. administration of MYR or 1-MT (suspended in MYR) caused local swelling around the puncture sites. However, no fever response or significant changes in feed uptake or body weight were observed.

At 8:00 a.m. on the fifth day, half of the pre-treated and control animals received a single i.p. administration of LPS with a dose of 50 µg/kg live weight (*Escherichia coli* O111:B4; Sigma-Aldrich, Deisenhofen, Germany) dissolved in 3 mL sterile endotoxin-free 0.9% NaCl according to a previous description [41]. The other animals were treated with an equivalent volume of NaCl. Twelve hours before LPS/NaCl application, the feed was removed to avoid an interference of feed uptake with TRP metabolism during the sampling period immediately after endotoxin challenge.

#### 4.2.1. Experiment 1: Effects of 1-MT on Inflammatory Response and KYN Pathway

Because one animal had to be excluded due to health problems, 47 animals were used in total in this experiment, distributed in the following treatment groups as described above: 1-MT + LPS (*n* = 12), 1-MT + NaCl (*n* = 12), MYR + LPS (*n* = 11) and MYR + NaCl (*n* = 12). Before every s.c. 1-MT/MYR application, blood was collected to analyse plasma concentrations of 1-MT and TRP metabolites. In addition, blood samples were collected immediately before i.p. LPS/NaCl administration (0 h) and at 1, 3, 6, 12 and 24 h after administration for analyses of 1-MT, TRP metabolites and cytokines (TNF-α, IL-10) in plasma. Blood sampling was carried out while pigs were in a supine position by anterior vena cava puncture with the whole procedure lasting less than one minute. Blood samples were collected in ice-cooled tubes containing EDTA or heparin and centrifuged at 2000× *g* for 10 min at 4 °C. The blood plasma was then stored at −80 °C until analysis. Before s.c. 1-MT injections and before blood samplings during LPS challenge, skin temperature was measured in the inguinal region using an infrared thermometer (ThermoScan IRT 4020, Braun, Kronberg, Germany).

For collecting tissues, six animals each from every treatment group were euthanized by an injection of T61 (Intervet, Unterschleißheim, Germany) at 6 and 24 h after LPS/NaCl. These times of tissue collection were chosen because they represent the temporal dynamics of IDO1 activity based on alterations of TRP metabolite concentrations as shown previously [14]. After euthanasia, liver, lung, muscle (Musculus deltoideus), adrenal gland, spleen, thyroid gland, heart, kidney and brain tissues were quickly removed. The hypothalamus, hippocampus, amygdala and prefrontal cortex were dissected from the brain, frozen in liquid nitrogen and stored at −80 °C. The stereotaxic atlas of the pig brain served as a reference [42]. Tissues were analysed for 1-MT and TRP metabolite concentrations.

#### 4.2.2. Experiment 2: Effects of 1-MT on Behaviour and Haematopoiesis

In experiment 2, a total of 48 animals were used. Animals were pre-treated with 1-MT or MYR and received either an LPS or NaCl administration in accordance with experiment 1. Blood samples were collected before every s.c. application of 1-MT/MYR, immediately before (0 h), and 6, 12 and 24 h after LPS/NaCl administration. Evaluation of sickness behaviour was conducted from 0 to 5 h after LPS/NaCl treatment. Differential leukocyte counts were evaluated at 0, 6 and 24 h after i.p. LPS/NaCl application.

### 4.3. Analyses

#### 4.3.1. Quantification of 1-MT and TRP Metabolites

The determination of 1-MT, TRP, KYN, KYNA and QUIN in EDTA plasma and tissues was performed using methods that have been previously described in detail [14,15] using an HPLC-system (Perkin Elmer, series 200, Darmstadt, Germany) and an API2000 tandem mass spectrometer equipped with an electrospray ion source (ABSciex, Darmstadt, Germany). The main quality parameters of between-day and within-day accuracy and precision as well as the analytical ranges and linearity (correlation coefficient, R) are provided in Table 3. The obtained results adhere to international recommendations [43,44]. As an indicator for IDO1 activation, the ratio of KYN and TRP (KYN × 100/TRP) was calculated [45].

#### 4.3.2. TNF-α and IL-10 Assays

The concentrations of TNF-α and IL-10 were analysed in duplicate in blood plasma using a commercially available pig ELISA kit (Invitrogen, Frederick, MD, USA) according to the manufacturer’s instructions. The sensitivities of the TNF-α and IL-10 assays were 3 pg/mL. The intra- and inter-assay coefficients of variation (CV) of TNF-α were 6.2% and 8.2%, respectively. The intra- and inter-assay CVs of IL-10 were 6.3% and 9.4%, respectively [46].

#### 4.3.3. Differential Leukocyte Counts

A blood smear was prepared followed by air drying. The object slide was then incubated for 2 min in May–Grünwald solution and washed with aqua dest, followed by an incubation in Giemsa solution (1:20) for 30 min. After washing with aqua dest, the slide was dried. To calculate the leukocyte distribution, a total of 200 leukocytes was counted using microscopy. The cell types were differentiated as lymphocytes, monocytes, basophiles, eosinophils and neutrophils with subdivision into neutrophilic myelocytes, band neutrophils and segmented neutrophils [47]. As a marker for early acute inflammation and physiological stress, the neutrophil to lymphocyte count ratio (N/L ratio) was calculated [33]. The N/L ratio is a biomarker reacting very early in the course of acute inflammation [34].

#### 4.3.4. Behavioural Observations

The behaviour of each animal was observed using scan sampling [48] every 5 min over a period of 5 h after i.p. LPS/NaCl application, resulting in 60 observations per animal. Thus, at the time of observation, individual behaviour categorized as symptoms of sickness, activity or inactivity was assessed for each animal (Table 4). A value of 1 was assigned to indicate the occurrence of a specific behaviour, whereas a value 0 was assigned to denote its absence. To evaluate the severity of sickness, the values of all sickness symptoms were summed for each observation point for each animal (minimum number = 0, maximum number = 5) and calculated as the mean number. Furthermore, an animal was characterized as active or inactive if the behaviour of the category was recorded at the time of observation. Then, the activity level was calculated as the percentage of the whole observation period of 5 h for each animal.

### 4.4. Statistics

Statistical analyses were performed using SAS software, version 9.4 for Windows (SAS Institute Inc., Cary, NC, USA). Descriptive statistics and tests for normality were calculated with the UNIVARIATE procedure provided with the Base SAS software.

Blood and tissue parameters and skin temperature were evaluated by ANOVA using the MIXED procedure with SAS/STAT software. In experiment 1, plasma TRP metabolites were evaluated before LPS/NaCl application. This model comprised the fixed effects pre-treatment (1-MT, MYR), sampling time (−84, −60, −36, −12 h), replicate (1, 2, 3) and their multiple interactions. The repeated statement in the MIXED procedure and a compound symmetry block diagonal structure of the residual covariance matrix were used with respect to repeated measurements on the same animal. For the plasma parameters and skin temperature from experiment 1 after LPS/NaCl application, ANOVA comprised the fixed effects pre-treatment (1-MT, MYR), treatment (LPS, NaCl), sampling time (0, 1, 3, 6, 24 h) and replicate (1, 2, 3), their multiple interactions, and the random effect sow and repeated statement (as described above). For evaluation of tissue parameters, data of both sampling times (6 and 24 h) were combined and ANOVA consisted of the fixed effects pre-treatment (1-MT, MYR), treatment (LPS, NaCl) and replicate (1, 2, 3), their multiple interactions and the random sow effect. For blood parameters in experiment 2, ANOVA comprised the fixed effects pre-treatment (1-MT, MYR), treatment (LPS, NaCl), sampling time (0, 6, 12, 24 h) and replicate (4, 5, 6), their multiple interactions and the random effect sow. The repeated statement in the MIXED procedure was used as described above.

To evaluate sickness severity during the 5 h observation period after LPS, the GLIMMIX procedure was applied using a Poisson model (model statement: distribution = Poisson, link = log) comprising the fixed effects pre-treatment (1-MT, MYR), treatment (LPS, NaCl) replicate (4, 5, 6) and their interactions. For the evaluation of activity in the 5 h observation period, the GLIMMIX procedure was applied using a logistic model (model statement: distribution = binomial, link = logit) comprising the fixed effects pre-treatment (1-MT, MYR), treatment (LPS, NaCl), replicate (4, 5, 6) and their interactions. Repeated measurements on the same animal were taken into account by the “_RESIDUAL_” keyword in the “random statement” of the GLIMMIX procedure using the autoregressive structure of the first order (type = AR(1)) for the block diagonal residual covariance matrix.

For the presentation of the results, the least square means (LS-means) and their standard errors (SE) were calculated and tested for each fixed effect in the models described earlier using the Tukey–Kramer procedure for all multiple pair-wise comparisons. Effects and differences were considered significant at *p* < 0.05.

## 5. Conclusions

The results from the present study in pigs indicate that repeated administration of 1-MT does not inhibit the LPS-induced increase of KYN/TRP ratio, which is used as a marker for IDO1 activation in vivo. Therefore, no evidence is provided that the 1-MT-induced effects in this study are related to a reduced IDO activity. Indeed, 1-MT increased the levels of KYNA prior to the LPS challenge, indicating a pre-treatment effect of 1-MT facilitating the degradation to one branch of the KYN pathway. The 1-MT-associated modulation of the LPS-induced haematopoiesis indicates an interference of 1-MT with inflammatory signalling pathways. This might be due to the activation of AhR by 1-MT and KYNA resulting in the transcriptional activation of several inflammation-associated target genes. Furthermore, the general increase in TRP by 1-MT may cause an impaired antimicrobial defence if applied in sepsis patients. These adverse effects of 1-MT should be considered in therapeutic applications of 1-MT, which is used in clinical studies in tumour patients with the goal of preventing IDO1-induced immune escape of cancer cells.

## Figures and Tables

**Table 1:** Pairwise comparisons of the pre-treatment 1-MT vs. MYR and the treatment LPS vs. NaCl in plasma using a Tukey–Kramer test.

	1-MT		MYR		SE	*p* Value		
	LPS LSM	NaCl LSM	LPS LSM	NaCl LSM		1-MT vs. MYR	LPS vs. NaCl	Interaction
TRP (µM)	127.38 ^A^	129.67 ^A^	115.63 ^B^	122.29 ^C^	1.10	<0.001	<0.001	<0.05
KYN (µM)	1.64 ^A^	0.99 ^B^	1.54 ^A,B^	1.17 ^A,B^	0.16	0.78	<0.01	0.40
KYN/TRP ratio	1.22 ^A^	0.83 ^B^	1.30 ^A^	0.98 ^A,B^	0.11	0.22	<0.001	0.69
KYNA (µM)	11.9 ^A^	11.5 ^B^	2.1 ^C^	2.1 ^C^	0.07	<0.001	<0.001	<0.01
QUIN (µM)	1.98 ^A^	1.83 ^A^	2.18 ^B^	1.98 ^A^	0.05	<0.01	<0.001	0.36

Concentrations of TRP, KYN, KYNA, Ratio (KYN × 100/TRP) are presented as LS-means (LSM) and SE. Within a row, values with different superscript letters differ with *p* < 0.05. 1-MT: *n* = 24, MYR: *n* = 23; LPS: *n* = 23; NaCl: *n* = 24.

**Table 2:** Pairwise comparisons of 1-MT vs. MYR and of LPS vs. NaCl in peripheral and brain tissues using Tukey–Kramer test.

	1-MT LSM	MYR LSM	SE	*p* Value	LPS LSM	NaCl LSM	SE	*p* Value
**TRP (nmol/g protein)**								
Amygdala	1094	1067	88	0.83	892	1269	88	<0.01
Prefrontal cortex	563	416	29	<0.001	477	502	29	0.47
Adrenal gland	1062	855	46	<0.001	926	992	46	0.22
Muscle	484	385	19	<0.001	397	472	18	<0.01
Lung	489	393	32	<0.05	396	486	31	<0.05
Liver	715	619	33	<0.05	597	737	32	<0.01
Heart	685	569	41	<0.05	569	685	39	<0.05
Hippocampus	224	178	11	<0.01	189	213	11	0.12
Hypothalamus	79	67	4	<0.05	70	76	4	0.19
Spleen	746	636	31	<0.05	687	696	32	0.84
Kidney	1337	1249	42	0.10	1211	1375	42	<0.01
Thyroid gland	459	495	30	0.31	469	485	29	0.67
**KYN (nmol/g protein)**								
Liver	604.86	590.86	35.62	0.64	586.74	608.98	34.99	0.48
Lung	123.16	156.84	21.97	0.29	180.15	99.85	22.08	<0.05
Spleen	12.83	11.45	1.79	0.58	18.28	6.00	1.80	<0.001
Hippocampus	19.69	19.74	1.78	0.98	19.50	19.93	1.78	0.87
**KYNA (nmol/g protein)**								
Lung	3.83	2.4	0.34	<0.01	3.34	2.90	0.005	0.30
Kidney	0.71	0.57	0.05	<0.05	0.65	0.63	0.05	0.80
Liver	5.84	4.75	0.46	0.06	5.64	4.95	0.47	0.23

Results of TRP, KYN and KYNA concentrations are presented as LS-means (LSM) and SE. 1-MT: *n* = 24; MYR: *n* = 23; LPS: *n* = 23; NaCl: *n* = 24.

**Table 3:** Analytic quality parameters for 1-MT, TRP and its metabolites.

Analyte	Accuracy (%)	Precision (%)	Range (µM)	Linearity (R)
1-MT	−7.8–4.0	2.3–7.0	2.3−69	0.9966–0.9992
TRP	−5.9–3.3	1.7–9.9	1.2–49	0.9986–0.9995
KYN	−9.5–13.5	3.9–14.5	0.24–24	0.9969–0.9990
KYNA	−9.9–15.7	6.8–14.9	0.01–0.32	0.9931–0.9946
QUIN	−19.8–18.4	7.6–19.8	18–359	0.9590–0.9957

**Table 4:** Categories and their related behaviours used for the assessment of sickness severity and activity.

Category	Behaviour
Symptoms of Sickness	Shivering; impeded respiration; vomiting; diarrhoea; circulatory insufficiency
Activity	Walking; drinking; employment with feed, bedding, trough, toy or piglet from neighbouring pen
Inactivity	Lying; sitting; standing without movement

## Abbreviations

IDO1	Indoleamine 2,3-dioxygenase
1-MT	1-Methyltryptophan
LPS	Lipopolysaccharide
TRP	Tryptophan
KYNA	Kynurenic acid
KYN	Kynurenine
QUIN	Quinolinic acid
MYR	Myritol
N/L ratio	Neutrophil to lymphocyte count ratio
TDO2	Tryptophan 2,3-dioxygenase
AhR	Aryl hydrocarbon receptor
CYP1A1	Cytochrome P450, family 1, subfamily A, polypeptide 1
GM-CSF	Granulocyte-macrophage colony-stimulating factor
iNOS	Inducible nitric oxide synthase

## Appendix B

### Appendix B.1. Supplementary Experiment

The potential interactions of 1-methyltryptophan (1-MT) and the amount of tryptophan (TRP) contained in 1-MT with aryl hydrocarbon receptor (AhR) and inflammatory pathways were evaluated in a primary cell culture of porcine lung fibroblasts. As a sensitive marker for AhR activation, the mRNA expression of cytochrome P450, family 1, subfamily A, polypeptide 1 (CYP1A1) was evaluated [40]. To examine the potential activation of inflammatory pathways by 1-MT, the mRNA expression of TNF-α, IL-8, granulocyte-macrophage colony-stimulating factor (GM-CSF) and inducible nitric oxide synthase (iNOS) was analysed.

### Appendix B.2. Methods

Lung fibroblasts were isolated from nine-week-old German Landrace pigs (*n* = 6) directly after slaughter according to the isolation method of human colonic lamina propria fibroblasts as described earlier [49]. Briefly, lung tissue was cut into 1-mm pieces and incubated for 30 min in Hank’s Balanced Salt Solution without Ca^2+^ and Mg^2+^ (Lonza, Basel, Switzerland) with 2 mmol/L EDTA (Sigma-Aldrich, Deisenhofen, Germany). The remaining tissue was rinsed and then digested for 1 hour at 37 °C with 1 mg/mL collagenase 1 (Merck, Darmstadt, Germany), 0.3 mg/mL DNase I (Roche, Heidelberg, Germany) and 2 mg/mL hyaluronidase (Sigma-Aldrich, Deisenhofen, Germany) in PBS. The isolated cells were cultured in DMEM (Lonza, Basel, Switzerland) containing penicillin (100 IE/mL), streptomycin (100 μg/mL), and amphotericin B (1 μg/mL) (Lonza, Basel, Switzerland) and 10% FCS (Biochrom, Berlin, Germany). Porcine lung fibroblasts were used between passages 3 and 4. 1-MT (L-isomer, purity 95%; Sigma-Aldrich, Deisenhofen, Germany) was dissolved in 1 N NaOH to a stock concentration of 1M. Since it is known that 1-MT contain 5% L-TRP, a TRP control was prepared by dissolving 50 mM L-TRP (Sigma Aldrich, Deisenhofen, Germany) in 1 N NaOH. For porcine lung fibroblast challenge, 1-MT was diluted in fibroblast cell culture medium to a concentration of 100 µM. For TRP, 50 mM L-TRP stock was diluted to a concentration of 5 µM. A NaOH solvent control was prepared by adding 1 N NaOH to cell culture medium to the same volume as actually used for 1-MT and L-TRP challenge. After a cultivation time of 3 h, fibroblasts were dissolved in 900 µL Trizol (Thermo Fisher Scientific, Waltham, MA, USA) per well and RNA was isolated using Direct-zol™ RNA MiniPrep (Zymo Research, Irvine, CA, USA) with subsequent quantification on NanoDrop ND-1000 spectrophotometer (NanoDrop, Peqlab, Erlangen, Germany). The amounts of mRNA transcripts in cultured fibroblasts were determined by real-time PCR using LightCycler^®^ 480 instrumentation (Roche Diagnostics, Mannheim, Germany). Reverse transcription was performed with 500 ng of total RNA using SuperScript III First-Strand Synthesis System (Thermo Fisher Scientific) with random hexamers and oligo (dT) primers. For gene-specific polymerase chain reaction (PCR), 12.5 ng cDNA were used in reaction mixtures containing 20 μM each forward and reverse primers (Table A1) and GoTaq^®^ qPCR Master Mix (Promega, Madison, WI, USA), according to the manufacturer’s instructions. Templates were amplified in duplicate after 2 min at 95 °C by 45 cycles of the following program: 15 s at 95 °C for denaturation and 1 min at 60 °C for annealing and extension. B2M was not affected by the applied treatments (variance 1.9%) between the analysed groups and therefore used as a housekeeping gene. The crossing points were analysed using LightCycler^®^ 480 software (Roche Diagnostics) and the 2^−ΔΔ*C*^^t^ method [50] was used to calculate transcript levels of each target gene.

**Table A1:** Primer pairs for real-time PCR.

Primer	Sequence Forward	Sequence Reverse
**CYP1A1**	GATCTCTTCAAGGACCTGAATCA	GCTGGATATTGGCATTCTCGTC
**TNF-α**	CACCACGCTCTTCTGCCTACT	GCTGTCCCTCGGCTTTGACAT
**IL-8**	GCTGTTGCCTTCTTGGCAGT	CTGCACCCACTTTTCCTTGG
**GM-CSF**	GCAGACTCGCCTGAACCTGT	CAGCAGTCAAAGGGGATGGT
**iNOS**	CTGCGTTATGCCACCAACAA	TTTCCAGCCCAGGTCGATAC
**B2M**	CGTGACTCTCGATAAGCCCAAG	GATTCATCCAACCCAGATGCAG
